# Assessment of frailty: a survey of quantitative and clinical methods

**DOI:** 10.1186/s42490-019-0007-y

**Published:** 2019-03-18

**Authors:** Yasmeen Naz Panhwar, Fazel Naghdy, Golshah Naghdy, David Stirling, Janette Potter

**Affiliations:** 1 University of Wollongong, Wollongong, Australia; 2 Illawarra Health and Medical Research Institute, Wollongong, Australia

**Keywords:** Frailty assessment, Sensor technology, Gait analysis, Balance assessment, ADL

## Abstract

**Background:**

Frailty assessment is a critical approach in assessing the health status of older people. The clinical tools deployed by geriatricians to assess frailty can be grouped into two categories; using a questionnaire-based method or analyzing the physical performance of the subject. In performance analysis, the time taken by a subject to complete a physical task such as walking over a specific distance, typically three meters, is measured. The questionnaire-based method is subjective, and the time-based performance analysis does not necessarily identify the kinematic characteristics of motion and their root causes. However, kinematic characteristics are crucial in measuring the degree of frailty.

**Results:**

The studies reviewed in this paper indicate that the quantitative analysis of activity of daily living, balance and gait are significant methods for assessing frailty in older people. Kinematic parameters (such as gait speed) and sensor-derived parameters are also strong markers of frailty. Seventeen gait parameters are found to be sensitive for discriminating various frailty levels. Gait velocity is the most significant parameter. Short term monitoring of daily activities is a more significant method for frailty assessment than is long term monitoring and can be implemented easily using clinical tests such as sit to stand or stand to sit. The risk of fall can be considered an outcome of frailty.

**Conclusion:**

Frailty is a multi-dimensional phenomenon that is defined by various domains; physical, social, psychological and environmental. The physical domain has proven to be essential in the objective determination of the degree of frailty in older people. The deployment of inertial sensor in clinical tests is an effective method for the objective assessment of frailty.

## Background

Frailty is a physical phenomenon that is pervasive in older people. In spite of a significant number of studies in this area, there is a plethora of definitions for frailty but no prevailing consensus. Some definitions are derived from studies of certain populations and some are formulated based on clinical assessment methods. The most common approach is to associate frailty with aging [1], comorbidity, disabilities and chronic diseases even though frailty is a physical condition and different from aging, comorbidity and disability [2–4].

There are certain other characteristics associated with frailty. Loss of muscle mass (sarcopenia), dilapidation of the human body’s physiological system, and cognitive impairments are also considered as markers of frailty [3, 5, 6]. Such conditions in older people are often associated with an increase in the risk of fall, hospitalization, mortality and morbidity.They also make the activities of daily living (ADL) less independent.

This paper is a survey of the major quantitative and clinical methods proposed in the literature to assess and measure frailty. Each method is briefly reviewed and its strengths and deficiencies are identified. The state of the art in frailty assessment is determined and, research gaps and future directions in this discipline are discussed.

## Assessment tools of frailty using qualitative methods / clinical frailty instruments

The topic of frailty is well examined in the medical literature; in particular in the field of Geriatric Medicine. Several clinical frailty instruments are routinely used by geriatricians as clinical assessment tools. The first and foremost development began when Fried and colleague [3] demonstrated that frailty was a clinical state different from aging and comorbidity. However, they identified some relationship between disability, comorbidity and frailty [3]. Consequently, Fried et al. [3] provided a new view of the concept of frailty, from which Rockwood and team [5] identified a new approach, based on the accumulation of deficits, with which to address the frail condition of aged people. Rockwood extended his basic model defining frail status in the range of one to seven to a new scale of one to nine. Despite an abundance of such clinical instruments proposed in the literature, there is no standardized tool for frailty assessment. Because of its multi-directional nature, one assessment method cannot always guarantee accurate results in every domain [7, 8]. Moreover, these domains range from physical to psychological, and social to environmental. In the following subsections, the most widely used methods for addressing frailty in older adults in clinical practice are reviewed. Some of the methods are derived from population-based studies and then expanded to geriatric assessment tools. The selected clinical frailty models and their methods studied in this review paper are shown in Table 1.

**Table 1:** Comparative Analysis of Clinical Frailty Instrument

	Model Type	Assessment Method	Frailty Components	Frailty Scale	Evaluation Criterion
1	Fried Phenotype [3]	Subjective and Objective	Weight Loss Weakness, Slow Walking, Low Physical Activity, Exhaustion	7-point Frailty scale Non-Frail = No Phenotypes, Pre-Frail= 1 or 2 Phenotypes, Frail = More than 3 Phenotypes	covariate adjusted logistic model and Kaplan Meir
2	Clinical Frailty Scale (CFS) [5]	Subjective	Comorbidity, Function Measures	7-point Frailty scale	ROC, Interrater Reliability, Pearson Coefficient
3	Jones [14]	Impairments, Comorbidity Disability	Subjective	13-Point Frailty Scale Mild = 1-7, Moderate = 7-13, Severe = >13	Interrater Reliability, Sensibility
4	Edmonton Frail Scale [13]	Subjective	Cognitive Impairment, Balance and Mobility, Cognition, Heath Status, Functional Independence, Social Support, Medication Use, Nutrition, Mood and Continence	Maximum Score : 17 Severe Frail = Highest Score, Non-Frail= 0	Interrater Reliability, Internal Consistency, Construct Validity
5	Tilburg Frailty Indicator [16, 17]	Subjective	Physical, Psychological, and Social	Maximum Score=15 Severe Level	Hierarchical And Logistic Regression Analysis Interrater Reliability, Internal Consistency
6	Groningen Frailty Indicator [20]	Subjective	Physical, Psychological, Social and Cognition	Scale Range (0-4) Non-Frail =0, Severe Frail= (institutionalization)	Spearman Rank Correlations, Multi-Variate Regression Analysis

### Fried phenotype

Fried et al. [3] propose a model for defining frailty. This model consists of five phenotypes of weakness or exhaustion, grip strength, ADL, weight loss and gait velocity. Exhaustion and ADL are measured by an interview and the other three parameters are quantitatively calculated over time. Of these five components, weight loss is proved to be the least significant in estimating the frail condition [9]. Chkeir et al. [10] use the balance assessment method along with the Fried phenotype to perform binary classification of frailty (Non-Frail or Frail). Hewson et al. [11] devised a monitoring system for frailty diagnosis by deploying the Fried phenotype model. They utilized an accelerometer for ADL and gait velocity, bathroom scale for weight loss, a grip ball for exhaustion and a dynamometer for grip strength. Since these five phenotypes are quantifiable, the frailty assessments methods developed in biomedical engineering research prefer the Fried model.

### Rockwood’s frailty accumulation scale

Rockwood et al. [5] propose a seven-point clinical frailty scale (CFS). The study was based on a cohort of 2305 older adults (= > 65 years) who participated in the Canadian study of health and aging over a period of five years. Rockwood and colleagues deployed three approaches in their research. The frailty scale has a range from one (robust health) to seven (complete functional dependency). The assignment of the frailty scale was based on responses to a set of questions asked by the physician. The frailty components considered in determining the frailty scales were multidimensional; education, cognitive impairments, dementia with falls, dementia with impaired mobility, and frailty index mean score. Seventy frailty components (markers) were identified. The presence of a number of frailty components in the subject determines their final frailty measures. The study was based on the deficiencies experienced by the subjects. Since the study could only help the geriatrician, and because of its subjective nature, the frailty scale proposed by Rockwood et al. does not play any significant role in the research area of biomedical engineering. Acquiring data for all seventy factors for all subjects proved to be a quite lengthy process. All seventy markers had equal weights in evaluating the frailty index; however, some of these markers did not contribute as well to the definition of frailty as did other markers, but had the same weighting. Because of the limitations, the Rockwood Frailty Model is scarcely utilized in the research by engineers for quantitative analysis of frailty

Clegg et al. [12] conducted a study to devise an electronic Frailty Index by employing a cumulative deficit model. Their approach was similar to that of the Rockwood Model because the approach accumulated the presence and absence of deficits (thirty-six deficits were included). Clegg et al. used a retrospective study by anonymizing e-health records. The results were classified into four categories: fit, mild, moderate and severe. The study was designed based on the coding of electronic clinical records for patients and proved to be an accurate predictive model. However, further study was required to validate the model. Since no geriatrician was involved in assessing the frailty level, no questionnaire-based analysis was used.

### Edmonton frail scale

The Edmonton Frail Scale (EFS), developed by Rolfson and colleagues [13] is used to assess the frailty status by evaluating nine components independently and then accumulating the result. The EFS components are cognition, functional performance, general health status, functional independence, social support, medication use, nutrition, mood, and continence. All of these components were acquired by a simple questionnaire. The functional performance was evaluated by a TUG (timed up go) test which analyzed balance and mobility. The maximum possible score recorded for an EFS was seventeen which indicates a severe frail state and the lowest score (zero), indicates that no sign of frailty is perceptible. Rolfson et al. assessed the validity of EFS by conducting an experiment with subjects aged over sixty five. All the patients had a one-hour comprehensive geriatric assessment (CGA) with a specialist geriatrician and, in addition, all CGAs were completely unsighted by EFS scoring. The EFS was highly correlated with the geriatrician clinical impression of frailty, age and medication. In-patients scored higher than outpatients did. The inter reliability, internal consistency and construct validity of EFS proved to be high. Less than five minutes was required to execute EFS. This approach was subsequently recognized as a good choice for non-geriatricians to conduct routine EFS assessments. However, this still requires further study and cross validation because of the limited number of participants considered in the evaluation process.Overall, EFS is a subjective approach.

### Frailty index-comprehensive geriatric assessment by Jones

Jones and colleagues [14] further developed the Frailty Index-Comprehensive Geriatric Assessment (FI-CGA) scale for determining the frailty level among older people. Jones et al. used ten components to calculate a frailty index. The ten components of FI-CGA are cognitive status, mood and motivation, communication, mobility, balance, bowel function, bladder function, instrument ADL and ADL, nutrition and social resources. Older people with mild frailty scored less than seven, moderate scores are between seven and thirteen and those having severe frailty scored more than thirteen. The study cohort consisted of 169 participants. In order to be eligible for this study, it was mandatory that all participants be already considered to be frail. This means that this approach is not able to distinguish between frail and non-frail participants. Non-frail or healthy subjects were not used as a reference to distinguish various frailty levels. Ritta et al. [15] conducted a study on the prediction of mortality of FI-CGA along with the other four models (Frailty Index, CSHA, Frailty phenotype, and CFS) over a one year period. The FI-CGA proved to be the second most discriminating approach. The authors concluded that comorbidity played an important role in determining mortality with respect to inherent frailty status.

### Tilburg frailty indicator

Through a more extensive study, Gobbens et al. [16, 17] developed the Tilburg Indicator (TFI) to identify the level of frailty of older people. The TFI was primarily predicated on three dimensions: physical, psychological and social. The associated study was conducted over a four-year period and involved 484 community-dwelling aged people with an average age of 80.3. The TFI measures frailty in the absence of disability. The physical domain here comprises eight components and four physical components (weight loss, grip strength, gait speed and weakness) are the same as for the Fried’s phenotype. Depression, nervousness, cognition and coping with troubles were components of the psychological domain, and the social domain was based on living alone, social activity and social support. Although, TFI was an integral approach for measuring frailty, the study was inadequate to determine the relationship among the three dimensions. The maximum TFI score is fifteen but the scoring levels of TFI were not defined according to any gradation of frailty (such as non-frail, frail, mild frail or pre-frail). In an aligned study, Pialoux et al. [18] conducted a survey of clinical instruments used for frailty assessment. They analyzed ten different frailty-screening tools in terms of reliability, construct validity, content validity, internal consistency and other parameters. According to their results, the TFI measure proved to be a potential screening tool for frailty measurement with a comprehensive statistical evaluation. The only problem identified was the additional time required to conduct a TFI assessment. Gianaira and colleagues [19] determined the frailty status of a range of individuals by analyzing their gait parameters and posture index and compared these with the derived TFI measures.

### Groningen frailty indicator

The Groningen Frailty Indicator (GFI) is a fifteen question self-reported questionnaire based clinical frailty scale having binary responses (either 0 or 1). It is focused on four dimensions of frailty definition i.e. cognitive, physical, psychological and social. The maximum score on the scale is fifteen that indicates an institutionalized level of frailty, and the minimum score is zero indicative of the absence of frailty. Any score above four, indicates the existence of frailty in older people. In a following study conducted by Peters et al. [20] who utilized the GFI indicator, an internal consistency of 0.68, a convergent validity in the range of 0.45 to 0.61 and a divergent validity ranging from 0.8 to 0.5 were achieved. The participants in question were both home residents and in-patient aged people.

## Quantitative assessment

Various methods proposed in the literature utilize a single modality to measure frailty as a quantifiable physical parameter. However, the outcomes of these methods cannot be generalized because there is insufficient sample data, one time (short-term) observation of subjects, and a lack of statistical analysis in the experimental work. The wearable sensors, force platforms, foot switches, bathroom scales, and cameras (Kinect) are all measurement tools deployed to evaluate and monitor frailty [9, 21–23]. A number of the tools however, are also used in conjunction with other clinical frailty instruments. Hewson et al. [11] utilizes accelerometer data to evaluate physical activity and gait velocity to determine the frail status based on the Fried criteria. Inertial sensors are also extensively used for ADL analysis, balance assessment and gait analysis of older people. The use of a Kinect sensor in gait analysis has gained attention of researchers for gait pattern recognition and classification [24, 25].

In the following sections, an analysis of methods for measuring frailty quantitatively is covered. Table 2 lists the studies and their approaches for quantitative assessment of frailty using sensor technologies.

**Table 2:** Overview of the studies conducted using Sensor Technology

	Author	Frailty Prameters	Data Analyses	Sensor	Frailty Model	Comparing Clinical Instrument
1	M.A Brodie et al. [27]	Stair Ascent (ADL)	Cohen’s Kappa, Four Fold Cross Validation	Pendant Device (Barometer +Accelerometer)	None	
2	J. Bellmunt et al. [28]	ADL + Physical + Social	Bland-Altman Analysis Rule-Based Approach	Raspberry Pi, Industrial Sensor	None	
3	D.J. Hewson et al.[11]	Fried Phenotype	None	Tri-Axial Accelerometer In Smart Phone, Bathroom Scale, Grip Ball	Fried Phenotype	None
4	E. Gianaria et al. [19]	Gait Patterns + Posture Index	Pearson Coefficient	Kinect	None	TFI
5	R. Jaber et al. [33]	Fried Phenotype	None	Modified Bathroom Scale, Grip Ball, Doppler Sensor	Fried Phenotype	None
6	A. Chkeir et al. [10, 34]	Balance + Fried Phenotype	Kolmogorove-Smirnov Test, Mann Whitney U Test	Balance Quality Tester, Dynamometer	Fried Phenotype	None
7	R. Ganea et al. [55]	Posture Kinematics	Nonparametric Statistical Analysis (Wilcoxon Matched Pair)	Inertial Sensor (Two Accelerometers + Gyroscope)	None	Tinette Test
8	A.G. Mercant et al. [57]	Trunk Kinematics	Nonparametric Statistical Analysis (Mann-Whitney) and Cohen’s D	IPhone 4 (Accelerometer + Gyroscope)	None	Fried Phenotype
9	A. Martinez-Ramirez et al. [42]	Gait Parameters	ANOVA,	Tri-Axial Inertial Orientation Tracker	None	Modified Fried Phenotype
10	W. Zhang et al. [30]	ADL (Chair Rise Peak Power)	Spearman Correlation And Pearson Correlation	Pendant Sensor	None	GFI
11	A.Martinez-Ramirez et al. [36]	Balance Test	Continuous Wavelet Transforms, PCA	Tri-Axial Inertial Magnetic Sensor	None	Fried Phenotype
12	B.R. Greene et al. [63]	Balance and Mobility (TUG, five times Si-St and Quiet Standing Test)	ANOVA, support vector machine Classifier	Inertial Sensor (Tri-Axial Accelerometer + Tri-axial Gyroscope)	None	Fried Phenotype
13	Y.C. Chang et al. [21]	Weakness, weight, Slowness and Reaction Time, Functional Reach Strength, Reaction Time Balance	Neural Networks, Sensitivity and Specificity	E-Pad(Membrane Sensor), E-scale(LED + Wireless Unit), E-chair (Pressure Sensor + wireless Unit), E-Reach (Ultrasonic Distance Sensor),	Fried Phenotype	none
14	M. Schwenk et al. [51]	Gait, Balance and Physical Activity	Multimodal Logistic Regression	Inertial Sensor	Fried Phenotype	
15	N. Millor et al. [59]	Range of Movement, Acceleration and Power from 30s Chair Stand Test and Gait Speed from 3m Walking	Decision Tree, ANOVA	Inertial Sensor	None	Fried Phenotype
16	N.N.Toosizadeh et al. [31]	Elbow Flexion, and Extension (ADL)	Statistical Analysis	Inertial Sensor		Fried Phenotype

### Frailty assessment using ADL and mobility

Activities of daily living (ADL) play a crucial role in the determination of the health status of aged people and are used extensively as measurement tools in clinical approaches. ADL typically comprise various self-care activities like walking, bathing, sleeping, and dressing. Among them, activities such as walking, getting out of bed or sitting on a chair define the functional mobility of a person [26]. Hence, such activities are considered more significant in revealing the underlying functional status of aged people and functional mobility is used as an assessment tool for risk of fall and frailty. There are several methods reported in the literature to assess and monitor ADL. In this review, however, the focus is on those assessment methods of ADL that focus on a determination of a subject’s frailty status and we only consider those ADL monitoring systems which can be instrumental in evaluating frailty.

As part of their ADL study, Brodie and colleagues [27] examined how aged people ascend stairs. In these trials, they utilized an unobtrusive pendant device that recorded the stair ascent activity of all subjects (aged 83 ±4). Three sensor derived parameters of intensity, variability and stability were determined by the recorded vertical ascent velocity and barometric data. The intensity parameter was found to be directly related to the subject’s movement and hence increased ascent variability and reduced stability is a marker of frail people as they adapt defensive ascent strategies. The results did not show a significant correlation between muscle strength and stair activity, however the stair ascent/descent is a physical activity related to lower extremity. Mounting the sensor on the lower body could have revealed more significant results to differentiate between the healthy aged people(non-frail) and less healthy aged people(pre-frail, frail).

Bellmunt et al. [28] also used ADL as one of the components of a 3D (ADL, social and physical) frailty model. The frailty model was developed using qualitative data of participants living in private and nursing homes. However, the length of the study was insufficient to validate the model and only eight subjects participated in the trial. The frailty model was developed using logical rule of inference on semantic data; hence, the results do not warrant broad acceptance of their proposed frailty model. Their approach can be more useful and reliable for developing a frailty model if quantitative data is acquired through use of sensors.

Hewson et al. [11] designed a prototype frailty model using Fried’s phenotype. They also used a tri-axial accelerometer embedded in a smart phone for monitoring the ADL and physical activity by accumulative acceleration. The simple accumulation of acceleration cannot justify the number of activities performed by participants, as various activities are detected by acceleration signal patterns. There was no performance evaluation (such as sensitivity or specificity) in their work.

Liu and colleagues [29] undertook a comparative analysis of the Fried frailty model and Kanas city cardiomyopathy questionnaire frailty model in patients undergoing transcatheter aortic valve replacement to evaluate frailty. Low physical activity proved to be the strongest factor in predicting frailty in both frailty assessment methods. However, the data was collected using questionnaire and performance measurement without using any sensors.

A person rising from a chair, or “chair rise” is another significant daily life activity associated closely with balance and mobility. In a series of three experiments utilizing an unobtrusive pendant sensor worn by the subject, a detailed study was conducted by Zhang et al. [30] to determine the relationship of the chair rise with the associated peak power required for this movement. In this work, the authors also conducted a comparative analysis of the experiments with aligned clinical tests of the same participants. The three clinical tests were TUG, the Groningen activity restriction scale and the GFI. The results revealed a negative correlation of “chair rise” peak power in ADL with the GFI. The some of activities were detected as chair rise while monitoring the subject for longer, which suggests that chair rise activity detection algorithm using peak power needs further validation. Eventually, correlation of GFI and chair rise peak power is also affected. However, these studies were limited to evaluating the relationship among the clinical tests and the “chair rise” peak power parameter only with the limited number of subjects. Further studies are required to obtain sensor-based parameters for chair rise activity to measure frailty in a community dwelling people.

N.Toosizadeh et al. [31] conducted a study to develop a frailty model by deploying inertial sensors on the upper extremity (forearm and upper arm). The frailty criterion was derived from Fried’s phenotype based on objective measurement of slowness, weakness, flexibility and exhaustion. The study protocol consisted of performing elbow flexions and extensions as fast as the subjects were able for twenty seconds. The speed and rise time estimation quantifies slowness, power and moment of elbow quantified weakness and speed reduction and jerkiness quantified exhaustion. The results indicated accuracy is higher (right arm) when data from sensors (fore Arm+upper Arm) is used. However, if the two forearm sensors are deployed only, the decrease in specify and sensitivity were trivial. Overall, their approach was unique and less time consuming for frailty assessment by deploying a minimum number of sensors mounted on the body. The upper extremity motion analysis can be significant for measuring the frailty quantitatively. However, the similar test protocol for flexion and extension of the knee should be also applied. A significant comparison can be made for acute measurement of frailty.

A prior study conducted by Ranasinghe et al. [32] reveals the limitations of using wireless and vision sensors for monitoring the ADL for older people. According to the authors, there are three key issues in monitoring the ADL in older people. Firstly, the use of sensor technology to analyze the ADL should be unobtrusive and agreeable to the older people. Secondly, privacy issues when longer monitoring of various activities in unconstrained environments is needed. Finally, there is need for a universal classifier to recognize various activities of different populations.

### Balance analysis using sensor

Balance analysis plays a significant role in fall assessment among older people. The balance assessment tool is not exploited much for diagnosis of frailty. Jabar et al. [33] developed a project that incorporated multiple techniques for analyzing the frailty in older adults using the Fried phenotype model and developed associated interactive software. However, this approach had only a minor focus on balance. Chkeir and colleagues [10, 34] applied balance assessment methods to assess the physical frailty of aged people. Chkeir et al. [10, 34] further proposed a method for determining physical frailty by assessing balance using the Balance Quality Tester (BQT). The measurements of center of pressure (CoP) along with the anteroposterior and mediolateral directional sway were obtained using BQT. The vertical ground reaction force, stabilization segment, and peak time were extracted from the BQT signal. A Kruskal-Wallis analysis was used to classify these signals into robust, pre-frail and frail categories. However, their reported work was at a preliminary stage. Here, there is also an accepted need to exploit additional balance parameters to establish a more robust relationship between balance assessment and frailty.

Bertolotti and colleagues [35] designed an inertial platform unit comprising a tri-axial accelerometer, tri-axial gyroscope and tri-axial magnetometer to measure the movements of subjects. The experiment was conducted in three stages. In the first stage, inertial measurement unit (IMU) data was acquired from the subjects when they were asked to perform various exercises. In the second stage, subjects were monitored for longer whilst performing their daily activities. In this last scenario, various units were worn on a subject’s body to obtain the whole-body kinematics. These exercises included quiet standing tests derived from a Berg Balance test, a Tinetti and a Balance evaluation systems test. Although the model developed was extensively designed for facilitating balance assessments, their analysis only considered a limited range of balance parameters. The center of mass(CoM) was obtained using an inverted pendulum model from the inertial platform unit. However, statistical analysis of CoM features computed by a Wii Balance Board and sensor was not extensive. The discussion lacks details about body sway and balance parameters while performing balance tasks. Monitoring of ADL movement was part of their investigation but it is not well delineated in their paper. Overall, this work reveals a strong correlation of their customized IMU with an existing balance board; however, this experimental setup needs further investigation before being deployed for risk of fall assessment and frailty analysis.

In [36] Ramirez and colleagues conducted a study to assess posture control by conducting an standing balance test amongst three different age groups: frail, pre-frail and healthy individuals. The associated data was extracted from inertial sensors and temporal and frequency components were obtained by using wavelet decomposition. In a feet-together, eyes-closed test, values of the resulting CoM sway area were observed to be significantly greater in the frail group. Overall, results showed that wavelet decomposition analysis of acceleration and orientation signal was useful in discriminating healthy subjects from frail and non-frail. Ramirez also conducted semi tandem test with open eyes and closed eyes. A tandem test or semi-tandem test requires more postural control and very frail people are at the risk of fall while performing this task. The tandem test revealed more functional balance capacity than does a quiet standing test in which subjects balance along the CoM as it requires ankle and hip strategies along with arm motion to stand steady. However, results reported in the paper did not discriminate well between the three groups and failed to explain postural control phenomena in tandem tests among frail and non-frail group. Results showed the frequency components of frail and pre-frail group were less distinctive. Therefore, wavelet decomposition is not a favorable alternative for comparing various frailty levels.

A. Chkeir et al. [34] conducted quiet standing balance test using a bathroom scale device named a BQT. The four parameters derived from CoP(XYZ) were namely rise time, stabilization duration (when subject is standing steady), stabilogram area and average velocity of trajectory. These derived parameters were correlated with Fried’s phenotype. Only two parameters rise time and velocity of trajectory, showed a higher correlation with the phenotype of grip strength and gait velocity in discriminating frail and non-frail subjects. It is evident from this study that a simple standing test for twelve second is not sufficient to assess the balance in the elderly as it does not take any additional force or strategy to maintain balance along the CoM for such a short duration. The use of inertial sensors on the pelvis can be a good choice to quantitatively assess the balance for short durations by extracting sway area from sensor data as it is more sensitive to human motion than is a bathroom scale [37].

Alternatively, Chang and colleagues [21] have employed five frailty markers in a neural network model that was used in conjunction with four developed sensor units referred to as eScale, eChair, ePad and eReach. The ePad unit was developed by integrating membrane sensors within a carpet to measure balance performance by step detection. The eReach unit was formed by deploying an ultrasonic sensor on a hanger to determine the forward functional reach of the subjects. Here, the frailty model depends on time and distance measurements recorded by the sensors. The eleven input to the neural network were not all sensor driven parameters, as they included temporal parameters such as reaction time and, time taken to track steps. The experimental setup, however, was not portable or cost effective; as it requires specialized sensor units and wireless modules. There was no statistical analysis to correlate the eleven parameters with frailty outcomes. Overall, a semi-objective approach was deployed to determine the binary level of frailty (non-frail and pre-frail).

The vision sensor based approaches are widely reported in the literature to address risk of fall assessments, balance assessments and postural assessments [38–40]; yet such sensors are rarely used to assess frailty. However, vision sensors are preferred choice when patients or subjects have aversion to body-worn sensors while performing the tasks [32].

In a related approach, Lv and colleagues [40] estimated the balance parameters using a Kinect v2 in conjunction with a Wii balance board. Various view angles of the Kinect sensors were explored. The balance test was a functional reach test in forward and lateral directions. The results validated the use of Kinect device as a clinical tool for balance measurement by using the CoM in healthy subjects. The clinical balance tests are primarily used for the aged people to evaluate balance for their risk of both fall and frailty assessment. These experiments were, however, only conducted with healthy subjects. Therefore, a more extensive validation of their work on older people is needed before the method can be deployed as a clinical tool. Another limitation of this work was that the estimation of the CoM by the Kinect device was highly dependent on the actual position and detection of a subject’s feet.

### Gait characteristics

Gait patterns are used as body signatures in personal identification and rehabilitation [41]. A gait cycle is initiated when one foot makes initial contact with the ground and ends when the same foot again makes contact with the ground. During the gait cycle, various gait features can be identified. The gait cycle is divided into a swing phase and a stance phase. A swing phase is when a foot is in air and a stance phase is when both feet are in contact with the ground. The duration of the stance and swing phases can be treated as temporal gait features. When both feet make contact with the ground, it is called a double support. It is observed that the duration of double support is greater in older people because of their balance impairment. This can be an intriguing feature for assessing the frailty.

#### Analysis of gait characteristics using inertial sensors

Bravo and colleagues [23] conducted a study of the walking patterns of aged people in their homes. These trials involved extensive walking exercises, where an accelerometer was used to measure disturbance of their balance. The variation in balance was considered an early indication of frailty. Martinex-Ramierz et al. [42] exploited the spatiotemporal and frequency parameters of such gait variables in their study. They used two classification models for frail, pre-frail and robust classes. The first model relied only on gait velocity and the second model included gait velocity, step regularity, root mean square of the acceleration signal and total harmonic distortion of acceleration signal. An important outcome of the author’s work was formulation of the relationship between irregular gait patterns and frailty using multiple variable approaches (frequency components) rather than using temporal gait velocity only. The location of inertial sensor is critical to extract meaningful gait parameters. The location of inertial sensors at the lower extremities, e.g the shin or the ankle position, can generate gait patterns in the vertical direction and reveal more meaningful kinematic parameters as gait activity depends on the movements of lower extremities [43].

Analyzing step detection or step counts is another method of measuring gait characteristics. It is evident that step counts or gait speed of frail older people is lower than for younger healthier people. Marschollek and colleagues [44] compared four different algorithms for step detection of healthy subjects and mobility impaired in-patients by mounting an accelerometer on the trunks of subjects. However, none of these algorithms produced satisfactory results for measuring gait characteristics in older people. Accuracy was identified as a major problem at lower gait speeds.

Capela et al. [45] developed a 2–6 min walking test as a smart phone application. The mobile phone was worn at the lower back during the test to give a precise analysis of gait parameters including foot strikes, number of steps, step length, and cadence. This experiment was also recorded by video to visually derive the actual number of steps, foot strikes, and turns. This smart phone application promises to be an alternative solution to the 2–6 min walk clinical tests in rehabilitation centers and aged care facilities, but the study requires further exploration by validating the algorithm on a larger number of cases. The use of smart phones to extract gait parameters can be cost effective; however, data suffers from noise element and requires the preprocessing of data to extract kinematic parameters.

Rahemi and colleagues [43] developed a frailty model using six sensor- derived gait parameters of Toe-off speed, mid-Swing speed, mid-stance speed, propulsion duration, propulsion acceleration and speed norm. The parameters were derived from the angular velocity signal of sensors attached on the right and the left lower shin of the subject. Rahemi et al. correlated the gait parameters with frailty classes (non-frail, pre-frail and frail) and Fried’ phenotypes to develop a quantitative frailty model using statistical analysis and verified their results by a single layer artificial neural network model. Fried phenotype such as slowness and weakness were highly correlated with propulsion duration and propulsion acceleration. The major outcome of their work was demonstration of accurate and quantitative frailty measurements using lower extremity motion analysis with minimum number of sensors. The results were encouraging development of an integrated wearable shoe sensor device for long term monitoring of patients under unconstrained and unsupervised environment to measure frailty objectively.

#### Analysis of gait characteristics using vision sensors

Gianaira et al. [19] used computer vision and the Kinect based methodologies to assess gait pattern and posture index. In their experiment, the subjects performed the TUG test. The temporal parameters of walking time, swing time and double support time were extracted from Kinect data. The torso angle was the only kinematic parameter extracted from skeleton data and did not prove to be relevant to frailty levels. The decline in the torso angle is an age related phenomena that can differentiate the healthy subjects from frail aged. Swing and double support proved to be more effective for measuring frailty [9, 19].

Using a Kinect system in a clinical set up, Prochazka and colleagues [46] had earlier conducted an experiment to similarly extract gait parameters of patients suffering from Parkinson’s disease for comparison with healthy age-matched people. A skeleton tracking algorithm was used for joint location. Gait speed and stride length were extracted from a subject’s gait. This study achieved 90% accuracy in differentiating gait patterns between the patients with Parkinson’s disease and participants from the healthy age group. The stride length and gait speed were found to be pivotal gait features for detecting frailty [9, 46]. In a subsequent experiment, Prochazka et al. [47] employed both the image data and skeleton data to recognize gait disorders in Parkinsonian patients. This latter work also included subjects with Parkinson’s disease and age-matched healthy groups. Normalized average stride length was measured in the gait analysis. Motion was continuously tracked during a 4 m walk by each subject but it did not play a significant role in the motion analysis of the individuals with Parkinson’s disease. Here the authors achieved an accuracy of 91.7% in recognizing the gait disorder. The study was limited to comparison of the gait characteristics but similar algorithms can measure the gait characteristics of the frail older people. The study can be further extended to investigate the motion patterns of Parkinson’s patients and frail older people.

A similar experiment was conducted by Hotrabhavanda et al. [48]. In their experiments, they exploited both the skeletal and depth data acquired from Kinect systems to observe and analyze three gait parameters, stride length, stride time and gait speed, from a TUG test and a Figure of 8 Walk (F8W). The frame rate of the Kinect system was used to indirectly evaluate the stride time. The segmentation of a depth image by body index frames can track up to six subjects. A unique identity code was generated by body index frames to recognize each person. This unique code was useful to identify the frail subjects from a physician while the physician walked with frail patients side by side. This identification feature using unique codes was not explored in their analysis as the authors conducted experiments on non-frail subjects. The experiment demonstrated that, overall, the depth model was more accurate than the skeletal model. However, results suffered from occlusions during the TUG and F8W tests and the sample size used in this experiment was very small. Moreover, the location and angle of Kinect sensor placement plays a crucial role in extracting the spatial parameters. The accuracy of temporal gait parameters obtained through the Kinect sensor is higher than the spatial parameters such as stride length.

Gait speed is one of the significant characteristics for determining the frailty status in older people [49]. Gait speed is determined manually with the help of various clinical tests (e.g. a 3-meter walk test or a 2–6 min walk test). Nagano et al. [50] proposed a new method to evaluate gait speed by using the coordinate transformation approach from image processing. The authors here compared their results with data obtained from a motion capture system. Since the experiment was semi-automatic, it required guided digitization of the position for the recognized subject in the scene. The offline processing of data was another deficiency of this approach. The method of calculating the gait speed was simple. This approach requires various modifications when dealing with larger populations.

Schwen and colleagues [51] analyzed the parameters of gait, balance and ADL to determine the significant markers of frailty using a number of inertial sensors. The parameters of gait, balance and ADL were extracted by utilizing various algorithms such as LEGSys and BalanSens. Here, statistical analysis was used to isolate the important outcomes for identifying the most discriminating factors of frailty. According to the author’s investigation, stride length, double support, walking duration and gait speed were the most discerning frailty markers.

#### Dual task gait analysis

Dual task gait analysis has been used to predict fall in frail older adults [52]. Beauchet and colleagues [53] conducted studies on 30 frail older adults, using two types of simple cognitive tasks of backward counting and verbal fluency. The classification of frailty level was also evaluated by the Tinetti frailty scale. An obvious variation was observed in the gait pattern, gait speed and lateral gait stability when aged people were asked to perform some simple cognitive task while walking [53].The gait parameters were extracted manually from video recording. Hence, no significant quantitative relationships among the gait kinematic parameters, cognitive impairments and frailty can be formed.

Martinez-Ramirez et al. [54] developed a model using gait-kinematic parameters to distinguish frail older adults from other similar older people and frail subjects who also had mild cognitive impairments. The participants performed the five meters walking test while wearing an inertial sensor unit mounted on their lumbar spine L3. Spatio-temporal and frequency gait patterns were analyzed for three conditions of each subject’s walk: at their own speed, walk with an additional verbal task and walk whilst challenged with an arithmetic task. The subjects were categorized as either frail, with mild cognitive impairments or as the control group (robust old) using the Fried phenotype frailty model. The statistical analysis revealed that step regularity and symmetry were crucial gait parameters to distinguish the frail and non-frail groups with mild cognitive impairments from healthy control subjects. In addition, it was identified that gait variably was the main predictive parameter for risk of fall assessment. However, depending on the individual’s intellectual faculties, not every cognitive task affects the gait patterns significantly. The analysis did not consider the relationship between the degrees of frailty with dual task gait performance. It is evident that gait analysis plays a crucial role in determining frailty [19, 23, 42] and cognition is one of the parameters used in various clinical frailty instruments [5, 13, 20]. Therefore, dual-task gait analysis using sensor (vision or inertial) technology appears to accurately predict the degree of frailty.

### Postural analysis

Postural analysis and body sway have played significant roles in determining the risk of fall in older people [37]. Frail older adults are vulnerable to fall and exhibit an anomalous postural stability. Irregular postural data patterns can help to differentiate the frail and non-frail classes of older people.

#### Postural analyses using sensor

Ganea and colleagues [55] conducted an experimental trial on 30 patients to determine their frailty by analyzing the kinematics of body posture during Sit-to-Stand (Si-St) and Stand-to-Sit (St-Si) tests. The experiment was carried out in two stages: quantitative analysis of Si-St and St-Si tests before and after rehabilitation (baseline) program. Subjects were assessed using Tinette Test. Frailty was assessed by observing variation in the body dynamics i.e. transition duration maximum trunk acceleration, trunk tilt, trunk angular velocity and a fractal dimension parameter. The three parameters of transition duration, maximum trunk acceleration and a fractal dimension indicated noteworthy differences between the baseline and after rehabilitation. This study still needs further clarification on about relationships between the variables of body posture and degree of frailty. In a subsequent experiment, a single inertial sensor was employed to determine the rate of postural transition among the frail aged people [56]. The experiment was conducted by employing Si-St and St-Si clinical tests. The participants were from groups of older frail adults and others who were non-frail healthy adults. The outcomes here suggest that the frail participants exhibited a decrease in the rate of postural transition, took longer to perform Si-St and St-Si tests and exhibited an overall smaller trunk tilt.

Galan-Mercant and Cuesta-Vargas [57]conducted an Extended Timed Get-Up-and-Go test in two clusters of frail and non-frail older adults using an accelerometer and gyroscope embedded in a smart phone. The Fried phenotype model was used to assess frailty at a clinical level for comparison. The authors probed the Extended Timed Get-Up-and-Go test in five stages of Si-St, Gait-Go, turning, Gait-come, and Turn-to-Stand-to-Sit. During the Si-St phase of Extended Timed Get-Up-and-Go, the y-axis accelerometer data showed a dynamic variation between the frail and non-frail groups. Overall, the experiment showed lower accelerometer values for frail older people. The relationship between different sub phases of TUG test parameters with frailty levels should be investigated. These statistical relationship can be useful in predicting the outcomes of frailty.

Millor et al. [58] analyzed data from a series of 30 s chair Si-St and St-Si tests performed by a group of pre-frail aged subjects. The Si-St transitions were studied in detail by dividing a complete cycle into three sub-phases of impulse, stand up and sit down. Five parameters of phase time duration, orientation, angular velocity (X-axis), linear velocity (Z-axis), and linear acceleration (Z-axis) within a cycle were analyzed for pre-frail subjects. Although, subjects were classified as pre-frail by the Fried definition of frailty, their analysis can be further extended for risk of fall assessment and frailty classification. In a subsequent study [59] Millor and colleagues studied gait velocity along with kinematic parameters of the chair stand test for frailty classification. The four kinematic parameters of orientation range in anterior posterior, maximum value of acceleration in the vertical direction, power in vertical direction during the impulse phase and Si-St were highly correlated in identifying three frail groups. The results indicate that, apart from gait velocity, other kinematics parameters could be used to exploit frailty classification using sensor data with machine learning. Frail subjects showed wider anterior posterior turns, lower maximum peak power and lower accelerations in the vertical direction. In this work, the relationship of kinematics parameters with frailty levels was evaluated using the Fried phenotype model.

Clarke et al. [60] further exploited 3D anatomical landmark data obtained from a Kinect system, in order to evaluate postural control. The lateral reach, forward reach and single leg standing balance tests were performed by young subjects with reflective markers on various parts of the body. The precise placement of markers can be a time-consuming process and may cause some irritation when markers are mounted on the skin. In this work, the advantages and disadvantages of deploying 3D marker based systems were discussed. However, no statistical analysis of postural stability from 3D vision data was performed. Overall, the approach proposed may not be an ideal method for the analysis of the postural control stability in older people as the markers may cause some discomfort to the subjects and prevent them from performing the balance tasks effectively.

## The relationship between fall and frailty

Falling is one of the consequences of frailty. On the other hand, a fall may bring about frailty among aged people. Risk of fall assessment is an extensive research area in geriatrics and in biomedical engineering. There are diverse methodologies proposed in the literature to deal with risk of fall assessment, fall prevention, and predicting fall events.

By utilizing sensor technologies such as inertial sensors and force platforms (static and dynamic) to analyze static postures and dynamic motion, Ghahramani et al. [61] conducted a detailed study of assessing the risk of fall in older people. The study investigated both the clinical and quantitative methods used to assess the risk of fall and analyzed their disadvantages but the study provided no references to frailty assessment.

Various sensory devices, such as inertial sensors, vision sensors or interactive tools like the Kinect-based systems, are employed for fall analysis in older people [62]. The monitoring of ADL, balance assessment and gait analysis are widely utilized approaches for risk of fall assessment and predicting the risk of fall. Most of these methods (ADL, balance and gait) are now used for frailty assessment. Brodie et al. and Bellmunt et al. have used ADL for frailty analysis, Gianaira et al. employed gait feature analysis for frailty detection, and Chkeir et al. [10, 34] examined balance using the Fried phenotype model.

Greene et al. [63] designed a support vector machine classifier to determine a frailty index utilizing sensor data and the fall history of subjects. The experimental process here comprised three tasks: a TUG test, a five times Si-St test and a quiet standing balance test. However, the classifier design and statistical analysis did not discover any significant association between the frailty index and risk of fall.

In a follow-up study by Simila [64], a predictive model for risk of fall assessment was developed by using gait variables measured by accelerometers. These results were compared with the clinical balance scales of Activities-specific Balance Confidence, Berg Balance Scale and Geriatric Depression Scale. The experiment was conducted on a small number of subjects and the results did not reveal any significant relationships between the gait variables and the clinical scales used. The Kinect system and balance platform were utilized in the baseline pre-assessment of risk of fall. However, Kinect data and balance board data were not used in post analyses. Two of the parameters of the Fried phenotype, muscle strength and gait speed, were also measured but there was no comparison between risk of fall assessment and frailty.

Ganea et al. [55] also sought to identify the relationship between frailty and risk of fall assessment. In this work, the authors investigated body posture analysis and performed Tinette tests. The posture analysis was explored from the data produced by inertial sensors mounted on the trunk. The outcomes, however, also failed to demonstrate a precise relationship between frailty and risk of fall assessment.

Kim et al. [65] developed a 3-link human body model comprising four joint locations of neck, hip, knee and ankle to distinguish human activities (walking, standing up and sitting down) with and without a fall. The kinematic parameters were distance and inclination between two center of gravity (CoG) points in the 3-link body model. The CoG points were the midpoints of two triangles formed by joint location of the neck, hip and knee (first triangle) and the hip, knee and ankle (second triangle). Both kinematic parameters were evaluated by IMU and Kinect sensor data. The results indicate that fall is detected when the distance of the CoG decreases and the inclination reaches zero. Results from the sensors indicate coherency between them. However, no statistical analysis was performed to evaluate and validate coherency in the results obtained with the different sensors, and the study did not show how many subjects participated in the study. This approach could be extended to study CoG patterns of frail older people and could be exploited for risk of fall assessment. However, there is need for an investigation to establish a concise relationship between frailty and fall in older adults.

## Results

Gait parameter assessment is effectively used in risk of fall assessments. The gait assessment is also employed for frailty measurement at a rudimentary level. Gait speed is one of the key parameters used in the Fried frailty model. Other gait parameters, like stride length, gait variability, and double support, can also be integrated with gait speed for frailty assessment. These parameters can be evaluated using inertia or visual data. The former approach is exploited well but less work is done using visual data to identify frailty. The gait parameters can be classified into three categories: temporal, spatio-temporal, and frequency components. Table 3 shows the list of gait parameters related to frailty assessment. A limited number of frequency components are studied for frailty assessment and few studies explore the correlation between a harmonic ratio and the total harmonic distortion with frailty [42, 54]. Most of the studies utilize a time analysis to identify gait patterns and gait related parameters, and frequency analysis tools are not used extensively. Only two studies propose a classifier model to assess the frailty level and compare the results with clinical tools. The remaining studies determine the correlation of various gait parameters with predetermined frailty levels determined using clinical tools.

**Table 3:** List of Gait Parameters for Frailty Assessment

	Gait Parameters	Studies	Device	Clinical Test	Frailty Assessment
1	Gait Velocity	M. Schwenk et al. [26], A. Martinez-Ramirez et al. [42, 54], E. Gianaria et al. [19], N. Milor et al. [59], B. Hotrabhavananda et al. [48], A. Dubois et al. [62], A. Prochazka et al. [24]	5 Inertial Sensor Unit Shank, Thighs And Lower Back [26], Tri-Axial Inertial Orientation [42], Kinect Sensor [19, 24, 48, 62], Single IMU on L3 [59], Tri-Axial Inertial Sensor Lumbar Spine (L3) Acceleration Signal(Vertical Direction Only) [54]	Walk of 4.5 m [26], 3m Walk Test [42], TUG [19, 48, 59], 3m Walk Test, F8W [48], 5m Walk Test [54]	Pre-Classified Using The Fried Phenotype [26, 59], Pre-frail, Frail and Robust [42], Correlation of TFI, TUG and Gait Parameters [19], Classified as Frail, Frail with Mild Cognitive and Robust [54]
2	Step And Stride Regularity, Approximate Entropy, Harmonic Ratio(HR), Total Harmonic Distortion	A. Martinez-Ramirezet al. [42] [54]	Tri-Axial Inertial Orientation [42], Tri-axial Inertial Sensor Lumbar Spine (L3) Acceleration Signal (Vertical Direction Only)[54]	3 m Walk Test [42], 5m Walk Test [54]	Pre-Frail, Frail and Robust [42], Classified as Frail, Frail with Mild Cognitive and Robust [54]
3	Gait Symmetry	A. Martinez-Ramirez et al. [42]	Tri-Axial Inertial Orientation [42]	3m Walk Test [42]	Pre-frail, Frail and Robust [42]
4	Gait Variability	A. Martinez-Ramirez et al. [54]	Tri-Axial Inertial Sensor Lumbar Spine (L3) Acceleration Signal (Vertical Direction Only) [54]	5m Walk Test [54]	Classified as Frail, Frail with Mild Cognitive and Robust [54]
5	Signal Root Mean Square (RMS) Value	A.Martinez-Ramirez et al. [42]	Tri-Axial Inertial Orientation [42]	3m Walk Test [42]	Pre-Frail, Frail and Robust [42]
6	Stride Length	M. Schwenk et al. [26], Hotrabhavananda et al. [48], A. Procházka el. [24, 46, 47], N.A. Capela et al. [45],	5 Inertial Sensor Unit Shank, Thighs and Lower Back [26], Kinect [24, 46–48], Smartphone [45]	Walk of 4.5m [24, 26], TUG and F8W [48], 2–6 min Walk Test [45], Walk 4m (back and forth) five Times [47]	Pre-Classified Using the Fried Phenotype [26]
7	Stride Time	M. Schwenk et al.[26], Hotrabhavananda et al. [48]	5 Inertial Sensor Unit Shank, Thighs and Lower Back [26], Kinect [48]	Walk of 4.5m [26], TUG and F8W [48]	Pre-Classified using the Fried Phenotype [26]
8	Double Support	M. Schwenk et al. [26] E. Gianaria et al. [19]	5 Inertial Sensor Unit Shank, Thighs and Lower Back [26], Kinect Sensor [19]	Walk of 4.5m [26], TUG [19]	Pre-Classified Using the Fried Phenotype [26],Correlation of TFI, TUG and Gait Parameters [19]
9	Swing Time	E. Gianaria et al. [19]	Kinect Sensor [19]	TUG [19]	Correlation of TFI, TUG and Gait Parameters [19]
10	Stride Velocity	M. Schwenk et al. [26]	5 inertial sensor unit shank, thighs and lower back [26]	Walk of 4.5m [26]	Pre-classified using the Fried Phenotype [26]
11	Cadence	N.A. Capela et al. [45]	Smart Phone [45]	2–6 min Walk Test [45]	
12	Dual Task Gait Patterns	A. Martinez-Ramirez et al. [54], O. Beauchet et al. [67]	Tri-Axial Inertial Sensor Lumbar Spine (L3) Acceleration Signal (Vertical Direction Only) [54],	5 m Walk Test [54], Walk 10 m and Perform Two Cognitive Tasks Dual Task [67]	Classified as Frail, Frail with Mild Cognitive and Robust [54], Pre-Classified using Speechley and Tinetti Criterion [67]
13	Foot Strikes,, Pelvis Acceleration, Number of Steps, Length Distance Traveled	N.A. Capela et al. [45]	Smart Phone [45]	2–6 min Walk Test [45]	
14	Number Of Steps	N.A. Capela et al. [45], O. Beauchet et al. [67]	Smart Phone [45]	2–6 min Walk Test [45], Walk 10 m and Perform Two Cognitive Dual Tasks [67]	Pre-Classified using Speechley and Tinetti Criterion [67]
15	Step Time	A. Dubois et al. [62], N.A. Capela et al. [45]	Kinect [62], Smart Phone [45]	2–6 min Walk Test [45]	
16	Step Length	A. Dubois et al. [62]	Kinect [62]		
17	Toe-off speed, Mid-Swing speed, Mid-stance speed, propulsion duration, propulsion acceleration and speed norm	H.Rahemi. [43]	Inertial Sensor [43]	Walking Test [43]	Pre-Classified using the Fried Phenotype [43]

Balance impairment is one of the adverse effects of aging in older people and increases the risk of falls and the level of frailty. Thus, in the context of frailty, there is a rationale for assessing balance parameters such as CoM, CoP and the exhibited sway area. A limited number of studies have related balance parameters with a frailty phenotype, but each of these still require validation. Moreover, their approaches are limited to the Fried frailty definition. The significant parameters studied using sensors and clinical tests to assess the balance in older people are summarized in Table 4. Transition duration is a very useful variable to determine postural stability. The higher value of transition duration and its variability are strong indications of postural imbalance and hence they can be potential markers of frailty. High frequency components during a standing tandem test have also proved to differentiate healthy subjects from pre-frail and frail subjects. However, frequency patterns are less discriminating in frail and pre-frail patterns.

**Table 4:** Features of Balance Analyses

	Studies/parameters used to assess balance	Device	Clinical Test	Frailty Classification	Purpose
1	A. Chkeir et al. [10, 34] Rise Rate, Average Velocity of Trajectory Obtained From Reaction Force and CoP Trajectories	Balance Quality Testers (Bathroom Scales)	None	Frail and Non-Frail	Established the Relationship Among the Balance Parameter with the Fried Phenotype
2	Chang et al. [21] Balance	ePad and eReach	None	Pre-frail and Non-frail	Developed Frailty Model Using Artificial Neural Network.
3	A. Martinez-Ramirez et al. [69] Sway Area, Signal Patterns	Tri-Axial Inertial Sensor Unit	Quiet Standing Balance Test	Yes Pre-Classified According to the Fried phenotype	High Frequency Components Associated With Frailty Syndrome
4	G.M. Bertolotti et al. [35] Body Sway and Trunk Kinematics Data	Customized Inertial Sensor Unit (Gyro, and, Accelerometer)	Selected Tasks Performed from Tinetti Tests, Balance Evaluation Systems Test and Berg Balance	None	Validating the Use of Newly Developed Unit Against Balance Board and Marker-Based System.
5	Z. Lv et al. [40] CoM	Kinect	Double Leg Stance, sttar excursion balance Test	None	Validating Kinect V2 for Balance Measurement
6	A. Nalci et al. [79] Variation in pixel events	Camera	UPST	None	Developed Vision Based Model To Assess Balance

There are several methods for analyzing ADL. Table 5 details a list of ADL studies for fall and frailty assessment. It is obvious from the table that a limited number of activities that indicate a correlation with functional mobility in older people have been studied and a limited number of studies have evaluated frailty by using parameters extracted from sensor data in daily life activity [27, 30, 31]. The short-term monitoring of ADL to evaluate frailty can provide the advantage of using multiple sensors simultaneously for data acquisition. Walking, sitting, and standing activities are thoroughly investigated for shorter term monitoring as they can be easily implemented using clinical tests such as TUG, Si-St, St-Si and functional tests.

**Table 5:** Activities studied for fall and frailty assessment

S.No	List of Activities	Purpose	Sensor	Methods	Monitoring Duration
1	Elbow flexion and extension [31]	Frailty assessment	Inertial Sensor	Statistical Analysis	Short term (20 s test)
2	Walking [11, 38]	Fall Detection [38], Frailty Model [11]	Camera [38], smart phone [11]	Semi-Supervised, Computer Vision [38]	Short Term
3	Standing [38]	Fall Detection	Camera	Computer Vision	Short Term
4	Lying [38]	Fall Detection	Camera	Computer Vision	Short Term
5	Stair Ascent [27]	Differentiate Stair Climbing Pattern for Frail and Athlete	IMU worn as pendent	Wavelet decision tree	30 mins
6	Chair Rise Transfer [30]	Correlating Chair Rise Power with frailty (GFI) and Clinical Tests (TUG)	Pendent sensor	Support vector machine and Statistical Analysis	One Week Under Semi Controlled Environment
7	Handwashing [80]	Prediction of Cognitive Status	Camera	Machine learning	Video Recorded for Trials in Controlled Environment

There is a logical relationship between risk of fall assessment and frailty in older adults. The older people with higher frailty levels are at a higher risk of fall. Fall can be an outcome of frailty and may lead to hospitalization and institutionalization. Tests such as the Si-St,TUG test and clinical tests using sensor technology to assess the risk of falls and frailty are similar.

## Discussion

The studies reviewed in this paper can be grouped into two categories. The first group develops a frailty model to classify the frailty level and validate the classification results through clinical methods. In this approach, machine learning algorithms are deployed to design a frailty prototype model. The frailty model is developed using various frailty markers and kinematic parameters. Machine learning is then applied to distinguish between frail and non-frail older people [66]. This approach is not well explored.

In the second group of studies, subjects are already classified according to clinical methods as frail. The experimental work is conducted to confirm the clinical results and to measure the correlation between the quantitative and clinical results using statistical tools.

### Gait analysis for frailty

Gait analysis is a well explored research area in rehabilitation and geriatrics. Nevertheless, direct implication of gait characteristics for examining frailty is not fully explored. However, in order to study gait characteristics for frailty assessment, well-defined exclusion or inclusion criterion of the subjects need to be considered. These may include pre-existing mobility disorders or the utilization of walking aids and can be considered as exclusion criterion in evaluation of the gait parameters for frail subjects.

Gait velocity is one of the phenotypes from the Fried model that measures the slowness. Gait velocity is a fundamental and well-studied marker of frailty in older people. The gait speed can distinguish the three different levels of frailty (non-frail, pre-frail and frail) with greater accuracy than do other parameters. For example, double support can be more intuitive in discriminating non-frail and pre-frail only. There are limited studies that indicate that the frailty classification can be improved if other gait parameters, apart from gait velocity, are also used in developing a classifier. The gait features extracted from trajectories can be intuitive in assessing the frailty levels. Further investigation is required to define a robust relationship between various gait parameters and frailty. Moreover, the visual gait patterns and inertial data can be combined to produce other intriguing features for identifying frailty levels. The gait parameters obtained from walking tests whilst the subject is engaged in a cognitive task (arithmetic or verbal) are not proven to be strong markers of frailty. Overall, gait speed is decreased in both groups of frail and non-frail during walking tests combined with cognitive tasks such as counting in reverse order [54, 67]. Careful selection of cognitive tasks can increase accuracy of identification of frailty, as it combines physical and cognitive domains of frailty.

It is common to use standard clinical tests in the experimental setup. The most commonly used clinical test for gait assessment is the TUG test with a walk (some two to six meters) in a straight line. A TUG test is typically divided into three stages: a Si-St transition, walk, and St-Si transition. The TUG test also takes postural transition and gait analysis into account. A TUG test has been investigated using vision, Kinect and inertial sensors [68] and has proven to be a potentially valid clinical test for assessing frailty. Another variation of this test includes walking in a figure of eight path and combining straight and turning maneuvers. It can be more intuitive for determining balance than is a straight walking test (TUG, 3-meter walk) as the subject needs to also navigate the prescribed figure of eight pattern. However, further investigation is required to validate this test before it is deployed in clinical settings and for frailty assessment. In addition, comparative studies of gait parameters obtained through the straight line and turning phases of this test can be conducted.

### Balance assessment for frailty

Various clinical tests, such as Berge Balance assessment, quiet standing test (with eyes open and with eyes closed), Tandem, Uni-pedal stance test, and functional reach test, are deployed for balance assessment. Few balance tests are scrutinized to evaluate frailty levels. Most of these tests are investigated with the help of sensor technology using IMU and optical sensors for risk of fall assessment [37, 69, 70]. The use of a Kinect sensor is validated to assess balance in the older people against the established standards of balance measurement such as force platforms and marker based Motion Capture Systems [71, 72]. The star excursion balance test, Y-balance test and uni-pedal stance test can be unsafe for frail aged people, as these tests require sufficient muscle strength and good control of body motion. Future researchers may investigate the relationship between aging, balance and frailty by carefully selecting balance assessment tests. In addition, data from two or more sensors can be integrated to form more composite patterns to distinguish frailty levels. The Si-St and St-Si are frequently used in clinical tests to assess the functionality of lower extremities for risk of fall assessment [73, 74]. The variations of these tests such as 30 s, Si-St and five times Si-St tests are also commonly deployed at clinics by geriatricians.

Frequency analysis of balance tests is not explored well for classification of frailty levels. Other kinematic parameters, such as phase time, angle ranges of role, pitch and yaw, angular velocity, and orientation data need further investigation to devise frail patterns using these kinematic variables.

### ADL and frailty assessment

A common approach to analyze ADL for frailty assessment is to use IMU data. The higher acceleration value the larger is the activity level [11]. In another approach, different activities and the number of times those ADL are performed are measured. The use of activity detection algorithm is perquisite in this case and adds another computational cost. The higher the rate of strenuous activities, the less frail is the subject. Both approaches require long term monitoring that can be achieved with the help of IMU and vision sensors.

The application of vision sensors for long term monitoring has many limitations, such as occlusion, privacy issues, sufficient illumination and detection of persons of interest. These limitations lead to computation cost increases in processing video signals, which makes use of vision sensors a less favorable choice. The IMU sensors are generally unobtrusive; however, their deployment on the human body for long time monitoring may cause some agitation for the older subjects. A solution to this problem is to utilize a smart phone, which is usually equipped with a least one inertial sensor, or use light weight sensors fabricated as a pendent-like ornament.

Frailty assessment in clinical settings using ADL (elbow flexion, chair rise, stair ascent) eliminates the additional need for accurate activity detection as selected activities are performed in a controlled environment. Greater focus can be made on extensive analysis to extract the quantitative parameters from sensors to assess the frailty quantitatively. As fatigue and weakness are considered to be frailty markers [3], it is suggested that choice of physical tasks, such as longer distance walks, used in the experimental work should involve more body movement and require some physical strength and be exercised frequently to evaluate the gait parameters. This makes it easier to quantitatively extract the kinematic variables such as trunk angle, sway area, and deviation of CoM as these variables are investigated as frailty markers.

### The risk of fall and other factors as outcomes of frailty

A plethora of studies have been conducted independently to assess the risk of fall and a selected number of studies have evaluated frailty as a quantitative variable. In a small number of studies, a correlation between risk of fall and frailty is performed [55, 63, 64]. The outcomes of these studies are not satisfactory as they fail to develop a quantitative relationship between the risk of fall and frailty.

The outcomes of frailty are not limited only to risk of fall. Low activity level and decline in cognition can be also visible results of frailty. The Rockwood frailty scale delineates the frailty score with subject’s activity level and it relates the frailty score with health outcomes, which mainly involve physical activity. The Rockwood frailty scale also considers the effect of dementia [5]. However, the Rockwood frailty score is evaluated subjectively and does not take any quantitative or kinematic parameter into account.

It is significant to measure frailty quantitatively so that intervention can be considered in anticipation of adverse outcomes in older people. It is also important to assess and explore frailty in multiple physical domains of ADL, balance and gait. A wide range of kinematic parameters (such as gait speed, postural sway, trunk tilt) and sensor derived parameters (power, rise time) should be extracted and through rigorous statistical analysis applied to this data. The correlation of these parameters with frailty should be determined. Further frailty assessment methods should be performed to analyze parameters such as gait speed, moment of inertia, and variance coefficient that are highly critical in discriminating the frailty levels.

## Future directions

Frailty is a multidimensional physical state. There are various research issues associated with frailty. The need to develop an accurate frailty model that can be used as a paradigm for frailty analysis is imperative. Another significant research question associated with frailty is prevention mechanisms. There are various methodologies, such as improving postural stability and mobility to prevent falls [75, 76], proposed in the literature. One of the mechanisms used for fall prevention is the use of exergame technology instead of conventional physical therapies or routine exercise methods [75]. This approach uses exercise based interactive gaming platforms such as the Nintendo Wii which makes use of inertial and pressure sensors and a Kinect system that uses its vision sensors [75]. Since, there is an underlying relationship between fall and frailty, exergames techniques can be utilized to investigate whether frailty is a reversible process. Similarly, virtual reality is another approach proposed in the literature for reducing the risk of fall [76]. It is relatively cost effective and so there is a trend to use virtual reality in rehabilitation and training programs for improving balance.

Frailty can be intervened through the rehabilitation process, which may include some regular exercise. In this paper, we have not discussed mechanism for frailty prevention as it is beyond the scope of this survey paper. The future researcher may consider technology based frailty prevention methods as a new research area.

The studies reviewed in this paper considered frailty classification by categorizing frailty into different levels such as non-frail, pre-frail/mild frail and frail. The clinical instruments such as the Fried phenotype or TFI are used as reference models for frailty definition. Because of its simplicity in clinical applications, the Fried phenotype is used extensively as a prototype model. Moreover, it is replicated as an objective tool with the changes in the methods (quantitative) to determine frailty parameters. It is important to develop objective measurement devices to predict frailty in terms of a scale similar to the Rockwood frailty index that can classify frailty levels up to seven or nine. However, objective implementation of the Rockwood frailty model is not possible because it is based on the accumulation of deficiency, which cannot be measured with objective sensor technology. In addition to developing a delineated scale to measure frailty, the frailty scale should also relate the risk of fall associated with every level of frailty. Therefore, the enhanced frailty scale can be employed as a predictive model (mild frailty can lead to moderate frailty with more dependency on physical activity) that can perform the risk of fall assessment or predict the risk of fall in aged people. It is advantageous to develop frailty prevention mechanisms to avoid activities that can cause injuries or hospitalization.

The clinical frailty assessment methods are protracted processes causing fatigue and, because of their instructive nature, do not involve much interest from a participant. Therefore, another area of potential research is to investigate interactive and less time-consuming frailty assessment methods that involve active participation of older people.

In motion analysis, one sensor mounted on the body produces limited number of kinematic features. The approach can become more robust and powerful if the analysis is based on data produced by a variety of sensors. Since these approaches can provide information on different manifestation of frailty, integration of the data produced by them can provide a more intuitive approach to quantify frailty. Further statistical analysis can be carried out to determine the sensitivity of sensor-derived parameters to the frailty level. The sensors used for motion analysis, such as optical sensors, inertial sensor units, force platforms and balance boards, are less intrusive, easy to deploy in the experimental work and efficiently acquire data. It is evident that fusion of data obtained from various sources (for example from inertial and Kinect systems) has produced better results in identifying data patterns [77, 78]. To integrate multiple sensor data at feature level or to fuse those at decision level, may increase the overall accuracy of a general classifier for identifying various frailty levels.

The absence of a publicly available dataset is a barrier to generalizing the results of frailty assessment using a proposed methodology. Every research group conducts its own clinical tests using sensor technology and produces their own data sets. However, these datasets exhibit specific demographic conditions and are not shared with others. Once these datasets are available to the research community, more focus can be made on data mining approaches to extract key features and to develop machine learning algorithms for frailty classification. Moreover, with the availability of various datasets, frailty models can be cross validated over different demographic conditions and so lead to a more generalized frailty model.

## Conclusion

An acceptable measurement or definition of frailty needs cross validation so that the definition can be adapted globally. The frailty measurement tools should be able to classify the frail status of older people and anticipate possible consequence of frailty such as risk of fall, hospitalization, and need of walking aids, with better accuracy.

Because of the complexity and multi-dimensional nature of frailty with links to several different domains such as physical, psychological, social and environmental, one assessment method cannot always guarantee accurate results. Moreover, the nature of these domains is heterogeneous. Frailty can be detected and measured quantitatively with more precision and accuracy in the physical domain. The quantitative methods employed to assess frailty make use of kinematic parameters such as gait velocity, postural sway, and chair rise peak power. These parameters are obtained from the physical domain and hence it could be asserted that the physical domain is significant enough to assess frailty objectively.

## Methods

**Fig. 1:**
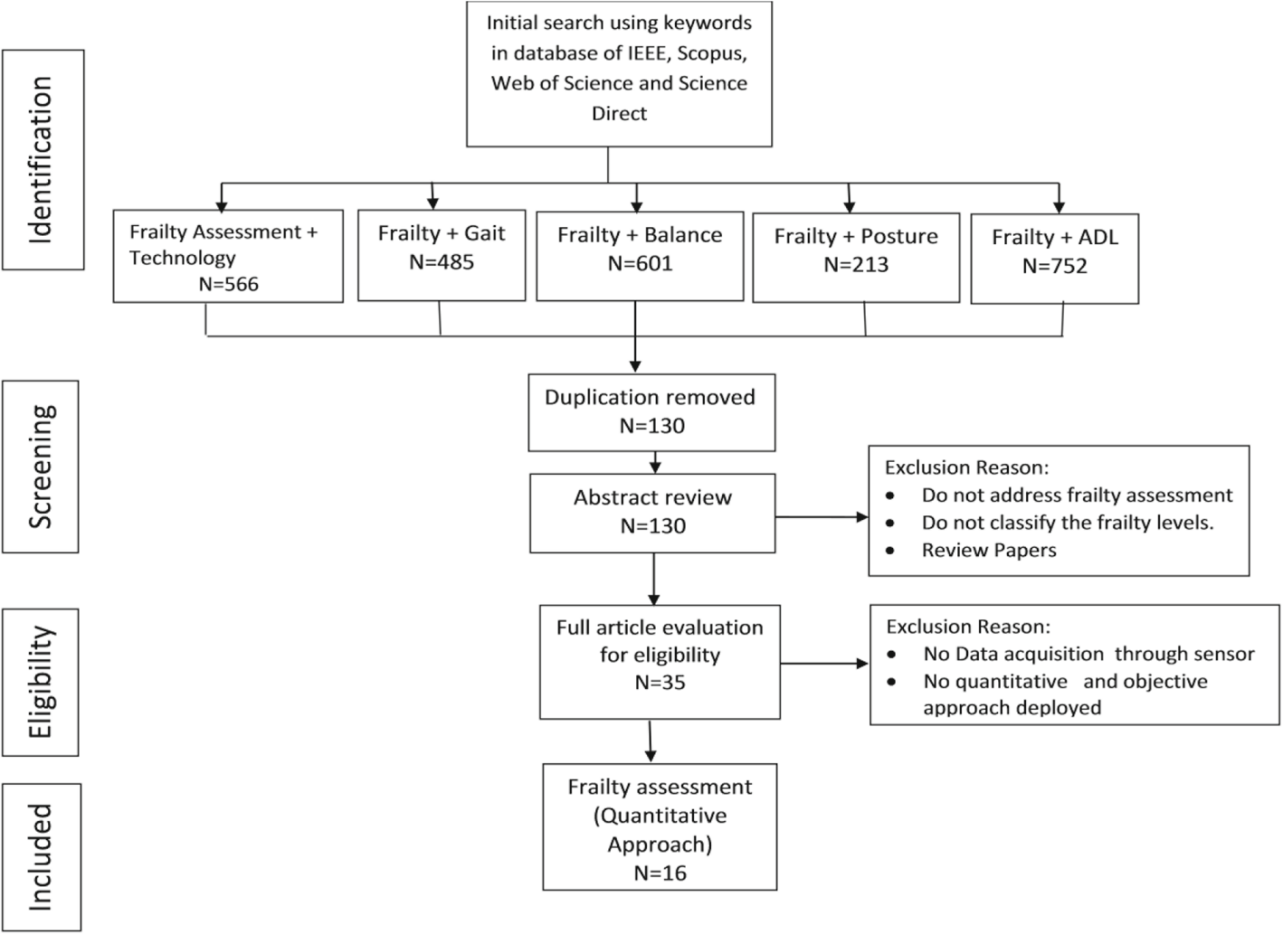
PRISMA flow diagram illustrating search strategy for paper on quantitative assessment of frailty

Two methods were developed to search the literature for quantitative and clinical studies of frailty. The process deployed for selecting appropriate papers about quantitative assessment methods is illustrated Fig. 1.

The databases used were IEEE, Science Direct, Scopus and Web of Science. The key words used in the search process were frail, frailty, assessment/analysis, older adults and synonyms of these terms. Other combinations of keywords used were “Gait analysis and Frailty”, “ADL and Frailty measurement”, “Sensor technology and Frailty”, and “Kinect and Frailty ”. The key words were applied to the search engines and any duplicate papers obtained in the search were removed. This resulted in 130 papers. The abstracts of these papers were examined to determine their relevance to the study, resulting in 35 potentially useful papers. The following inclusion criteria were developed and applied to papers

1. The research articles published in the English language.
2. The research papers published after the year 2001.
3. The research articles focused on frailty assessment methods for older people (above the age of sixty five) and that were gender neutral.
4. The studies were based on subjects who were not suffering from any specific disease (e.g. Alzheimer, Parkinson).
5. The data used in the study was produced through experimental work.
6. The length of the study and amount of data was sufficient to generalize the outcomes.
7. Frailty assessment methods were clearly defined.
8. The statistical analyses applied to the data extracted from experiments were appropriate and yielded the results in terms of frailty classification or frail status.

There were only sixteen papers that fully satisfied the quantitative assessment criteria and considered in the final stage of the review process.

The issue of frailty is well explored by the clinicians and researchers in the health sector. There are various studies described in the literature that use clinical methodologies to obtain information on the degree of frailty of a subject. The key words deployed to search the qualitative methods were frail, frailty, clinical assessment/tools/instruments, and older adults/ aged people. The keywords were searched in database of Web of Science, Scopus and Science Direct. A scanning of the literature in this area suggested that a cut off criterion for citations was 150 was appropriate. The other inclusion criteria were papers aimed at developing clinical frailty instruments and that could be implemented at the clinics. In addition, clinical instruments were referred or used as reference frailty model by quantitative studies. The data obtained was further interpreted and assessed by the geriatrician. Six clinical instruments were included in this study.

**Fig. 2:**
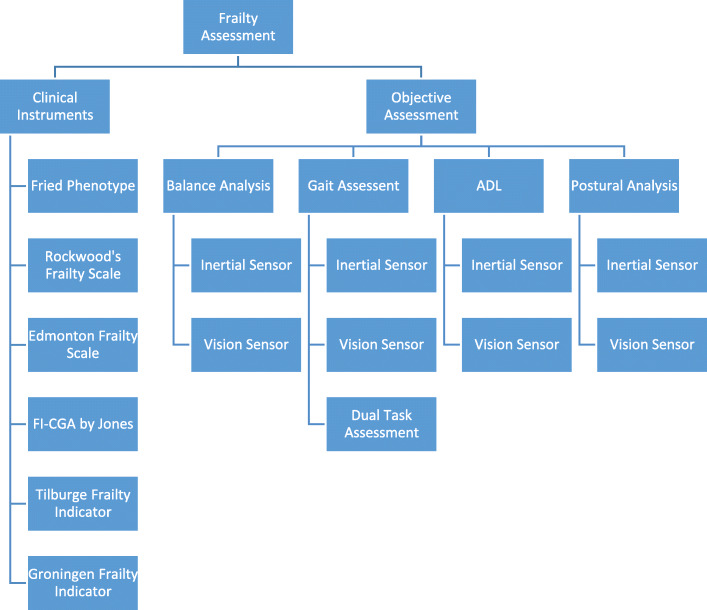
Quantitative and Qualitative assessment methods

The structure of the paper reflects the search process deployed. Accordingly, the content is presented in the two major sections of clinical methods and quantitative approaches using sensor technology. The section on quantitative assessments methods is further subdivided into categories of ADL, Gait Assessment, Postural Analysis and Balance Analysis as illustrated in Fig. 2. The applications of various sensor instruments and the possible combination of instruments for more accurate assessment of frailty in older people are discussed.

The section on qualitative methods covers the six clinical instruments of Fried Phenotype, Rockwood’s Frailty Scale, Edmonton Frailty Scale, Frailty index comprehensive geriatric assessment by Jones, Tilburg Frailty Indicator, and Groningen Frailty indicator.

## Abbreviations

ADL: Activity of Daily Living
BQT: Balance Quality Tester
CFS: Clinical Frailty Scale
CGA: Comprehensive Geriatric Assessment
CoG: Centre of Gravity
CoM: Centre of Mass
CoP: Centre of Pressure
EFS: Edmonton Frail Scale
F8W: Figure of Eight Walk
FI-CGA: Frailty Index Comprehensive Geriatric Assessment
GFI: Groningen Frailty Indicator
IMU: Inertial Measurement Unit
Si-St: Sit to Stand
St-Si: Stand to Sit
TFI: Tilburg Frailty Indicator
TUG: Timed Up and Go
